# Preliminary effects of a regional approached multidisciplinary educational program on healthcare utilization in patients with hip or knee osteoarthritis: an observational study

**DOI:** 10.1186/s12875-018-0769-7

**Published:** 2018-06-06

**Authors:** Aniek A. O. M. Claassen, Henk J. Schers, Sander Koëter, Willemijn H. van der Laan, Keetie C. A. L. C. Kremers-van de Hei, Joris Botman, Vincent J. J. F. Busch, Wim H. C. Rijnen, Cornelia H. M. van den Ende

**Affiliations:** 1 Department of Rheumatology, Sint Maartenskliniek, PO Box 9011, 6500 GM Nijmegen, The Netherlands; 2 Department of Primary and Community Care, Radboud University Medical Center, Nijmegen, The Netherlands; 3 Department of Orthopaedic Surgery, Canisius Wilhelmina Hospital, Nijmegen, The Netherlands; 4 Stichting Gezondheidscentrum De Kroonsteen - De Vuursteen, Nijmegen, The Netherlands; 5 Department of Orthopaedic Surgery, Sint Maartenskliniek, Nijmegen, The Netherlands; 6 Department of Orthopaedic Surgery, Radboud University Medical Center, Nijmegen, The Netherlands; 7 Department of Rheumatology, Radboud University Medical Center, Nijmegen, The Netherlands

**Keywords:** Patient education, Hip, Knee, Osteoarthritis, Self-management, Consistent information, Multidisciplinary

## Abstract

**Background:**

Providing relevant information on disease and self-management helps patients to seek timely contact with care providers and become actively involved in their own care process. Therefore, health professionals from primary care, multiple hospitals and health organisations jointly decided to develop an educational program on osteoarthritis (OA). The objective of the present study was to determine preliminary effects of this OA educational program on healthcare utilization and clinical outcomes.

**Methods:**

We developed an educational group-based program consisting of 2 meetings of 1.5 h, provided by a physiotherapist, a general practitioner (GP) and orthopaedic surgeon or specialized nurse. The program included education on OA, (expectations regarding) treatment options and self-management. Patients were recruited through searching the GPs’ electronic patients records and advertisements in local newspapers. At baseline and at 3 months follow-up participating OA patients completed questionnaires. Paired-sample t-tests, McNemar’s test and Wilcoxon Signed-Rank test were used to estimate the preliminary effects of the program.

**Results:**

A total of 146 participants in 3 districts attended the sessions, of whom 143 agreed to participate in this study; mean age 69.1 years (SD10.2).107 (75%) participants completed both baseline and follow up assessments. The proportion of participants who had visited their GP in the 3 months after the program was lower than 3 months previous to the program (40% versus 25%, *p*-value 0.01). Also, we observed a decrease in proportion of patients who visited the physio- and exercise therapist, (36.1% versus 25.0%, *p*-value 0.02). Both illness perceptions and knowledge on OA and treatment options changed positively (Δ-1.8, 95%CI:0.4–3.4, and Δ2.4, 95%CI:-3.0 - -1.6 respectively). No changes in BMI, pain, functioning and self-efficacy were found. However, a trend towards an increase in physical activity was observed.

**Conclusions:**

Our results show that a multidisciplinary educational program may result in a decrease in healthcare utilization and has a positive effect on illness perceptions and knowledge on OA due to clear and consistent information on OA and it treatment options.

**Trial registration:**

Netherlands Trial Register (NTR5472). Registered 22 September 2015.

**Electronic supplementary material:**

The online version of this article (10.1186/s12875-018-0769-7) contains supplementary material, which is available to authorized users.

## Background

Osteoarthritis (OA) is the most prevalent form of disability of posture and movement worldwide [1]. OA of the hip and knee is characterised by pain and stiffness which can impair daily functioning, and decrease physical activity [2]. This physical and accompanying mental burden influences the quality of life in patients with OA. Although there are no curative treatment options for OA, multiple effective non-surgical and surgical treatment options for reducing pain and improving movement ability and quality of life are available [2, 3].

International guidelines recommend a combination of pharmacological and non-pharmacologic modalities as primary approach for hip or knee OA [2–4]. Non-pharmacological treatment modalities include psycho-educational interventions to improve self-management, physical activity and exercise therapy, and weight reduction. Recommended pharmacological treatment consists of the use of acetaminophen (paracetamol), the use of non-steroidal anti-inflammatory drugs (NSAIDs) or, when the patient is not responding satisfactorily to oral analgesic/anti-inflammatory agents, intra-articular injections [2]. Once non-surgical treatments become unsuccessful, joint replacement surgery is a cost-effective procedure that can be considered for patients with severe symptoms [3]. However, joint replacement surgery is advised to be postponed as long as possible, as the lifespan of prostheses are limited [2] and the results can vary [4].

In recent years the total number of hip and knee replacement surgeries increased with 50 and 196% respectively, especially in the age group of 75–85 years [5]. Possible explanations for this overall increase are ageing of the population and increase in obesity resulting in more people suffering from symptomatic OA. Despite recommendations, conservative treatment modalities in hip or knee OA are underused [6, 7] while timely usage of these treatment modalities is advocated [8] and may prevent untimely surgery.

The underuse of conservative treatment can be caused by healthcare providers related barriers for recommending conservative treatment modalities. Research shows that outcome expectations about conservative treatment options differ widely among healthcare providers and the confidence in competencies of other healthcare providers is low [9–11]. As a result, patients with OA may not receive consistent information about effective, conservative treatment options. Receiving conflicting information is found to be associated with undesirable outcomes like non-adherence to treatment [12, 13]. Therefore, information on treatment options and strategies should be disseminated from a joint perspective of healthcare providers.

In addition, patient related factors might also influence the use of treatment modalities. Some patients are not aware of what they can do themselves and what conservative treatment options can be offered for their OA [14]. Providing relevant disease-related and self-management related information helps patients to become actively involved in their own care process [15]. Moreover, negative beliefs or unrealistic thoughts about different treatment modalities by patients might also influence the choice of treatment [16]. A recent systematic review showed that OA patients have a negative attitude towards the efficacy of conservative treatment and tend to prefer surgical treatment [17]. This emphasises the importance that patients are aware of benefits as well as possible disadvantages of both conservative and surgical treatment options, in order to have realistic expectations [4].

During a regional conference in the area of Nijmegen, the Netherlands, healthcare providers from different disciplines involved in the care for people with OA decided to develop a patient educational program with a multidisciplinary approach to tackle above outlined barriers for suboptimal care.

The aim of this program was to increase patients’ knowledge on OA, to stimulate self-management, to discuss benefits and disadvantages of treatment options, to promote the stepped care approach of treatments [8] and to provide consistent answers to frequently asked questions by patients. The objective of the present study was to determine preliminary effects of this OA educational program on healthcare utilization (HCU) and clinical outcomes.

## Methods

### Design and setting

An observational pilot study was performed in three districts in the Nijmegen area, the Netherlands, to evaluate a knee and hip OA educational program at baseline, and 3 months after finishing the course. In the period of October 2015 – March 2016, the program was organized 11 times (3–4 times per district). According to the Central Committee on Research involving Human Subjects (CCMO), this type of study does not require approval from an ethics committee in the Netherlands. This study was approved by the local Medical Research Ethics Committee, region Arnhem-Nijmegen (protocol number. 2015–2024).

### Study population

Patients were eligible for the program when they were aged 18 years or older and had a clinical diagnosis of OA in the knee or hip (diagnosed by a general practitioner (GP) or medical specialist). Exclusion criteria were inability to read or understand the Dutch language, and previous joint replacement surgery. A maximum of 20 people (including patients and their partner or other significant person) could participate in each of the 11 planned programs, in order to facilitate group interaction. We aimed to include a total of 110–132 patients with knee or hip OA (10–12 patients per program).

### Procedure

GPs’ and physiotherapists in the three different participating districts and several orthopaedic surgeons in de region were informed about the objectives, background and content of the study. They were asked to offer eligible patients a flyer with information about the knee and hip OA educational program. Additionally, in each district the GPs also invited patients with an already known OA diagnosis by mail. In order to minimize selection bias we selected all patients with a diagnosis code for hip or knee OA in the GP’s information system. GPs manually excluded patients who already had undergone joint replacement surgery or were not capable to understand the Dutch language. Moreover, an advertisement was placed in local newsletters and a local newspaper to invite patients. Once registered, a researcher checked eligibility of those patients.

After registration for the program, eligible patients received a letter with information of the study. By filling in a reply-card, patients could sign up for the program in their district. Participants received an additional information letter and an informed consent form, accompanied by a questionnaire on baseline characteristics and outcome parameters by mail, two weeks prior to the start of the course (T0). Three months after finishing the course, participants received a second questionnaire (T1) to assess the outcome parameters again.

### Intervention

The organised knee and hip OA educational program consisted of two 1.5-h meetings. The program was led by a physiotherapist and a GP, both working in the district where the program was held. Additionally, an orthopaedic surgeon or orthopaedic nurse practitioner and when available a public health advisor attended the program. One of the healthcare professionals in each of the carried out meetings was part of the research team. They were asked to approach healthcare providers in their own district to help them carrying out the meetings.

The educational program was developed by an expert group working in the field of OA. The expert group consisted of 2 orthopaedic surgeons, 1 rheumatologist, 1 nurse practitioner, 3 physiotherapists, 1 GP and 2 physiotherapist-researchers. First an inventory of frequently asked questions (FAQs) about OA was made among local health professionals. Second, a prioritising exercise was used among OA-patients and health professionals to determine the most important FAQs. Finally, the expert group discussed and formulated answers to the 20 most important FAQs until consensus was reached. A detailed description of the process of inventory and prioritising of FAQs is described in Additional file 1. The content of the program was based on this structured inventory of informational needs and on consensus-based information addressing those needs. The FAQs and answers were incorporated in the course material. In line with current guidelines on education for patients with knee or hip OA [18], the program consisted of information on: OA and its disease course, evidence based tailored conservative treatment in a stepped-care format [8], and surgical treatment options. Moreover, education was given on outcome risks of treatment options and expectation management. This information provided patients with knowledge on where to find the (treatment) help they needed, at the time they needed it, with the appropriate expectations about this treatment. Additionally, the program included information on regional options to enhance self-management and physical activity, tips, practical assignments and mottos on OA.

To support the information given during the course, participants received a booklet consisting of information, monitoring forms, course handouts, the 20 FAQs, a pedometer and a list of useful websites, mobile applications and contact information of organisations.

### Data collection

#### Baseline data

At baseline, patients’ characteristics were collected on: age, gender, the number of important comorbidities (ranging from 0 to 15) according to the Dutch Arthritis Impact Measurement Scales [19], living situation (alone / living with partner and/or family), education (low / high), ethnicity (native / foreign), employment (workless/paid work), duration of symptoms (< 1 year / 1–5 years / 5–10 years / > 10 years) and location of OA (hip and/or knee), and number of painful joints (including hip, knee, neck, back, shoulders, elbows, wrists, hands, ankle and feet).

#### Measurement instruments

Outcome parameters at baseline and 3 months follow-up were HCU, pain medication use, pain and functioning in daily living, illness perceptions, patient activation, knowledge, physical activity and patient satisfaction with the course.

HCU was assessed with a self-developed questionnaire. Patients were asked which healthcare providers they visited in the preceding 3-month period related to their hip or knee symptoms (yes/no) and to indicate the number of visits to these healthcare providers.

In addition, to record the use of pain medication, participants were asked if they used (yes/no) pain medication (paracetamol / non-steroidal anti-inflammatory drugs (NSAID) / other (i.e. tramadol, morphine)) in the past 3 months regarding their hip or knee OA.

To calculate BMI (weight/height^2^) weight and height were self-collected.

Two subscales of the Western Ontario McMaster University Index of osteoarthritis (WOMAC) were used to assess pain and limitations in functional activities. The WOMAC is a 24-item questionnaire, subdivided in 3 subscales: pain, stiffness and physical functioning [20]. WOMAC pain and physical functioning subscales were calculated and presented as normalized scores (0 to 100, with higher scores indicating less pain and better functioning).

Participants were asked to fill out the Dutch General Self-efficacy Scale (GSES) to measure self-efficacy [21]. The GSES has 10 items of which a total score can be calculated ranging from 10 to 40. With higher scores indicating higher self-efficacy.

The Brief illness perception questionnaire (IPQ) is a 8-item scale and was used to measure illness perceptions [22]. It measures patient’s cognitive and emotional perceptions with respect to their OA. The maximum score on the Brief IPQ is 80, with higher scores reflecting more threatening view of the OA.

To assess patient activation, defined as patients’ knowledge, skill, and confidence for self-management, the Patient Activation Measure (PAM-13) was used [23]. A total score can be calculated ranging from 13 (low confidence for managing own health and healthcare) to 52 (high confidence for managing own health and healthcare).

Physical activity was measured using the Short Questionnaire to Asses physical activity (SQUASH) [24]. The SQUASH consists of three main questions (days per week, average time per day and intensity) per activity-category (i.e. commuting activities, leisure-time and sports activities, household activities, and activities at work and school). A total activity score in min/week was calculated.

For the WOMAC, GSES, IPQ, PAM-13 and SQUASH a change of 20% was considered clinically relevant.

Based on identified frequently asked questions on OA in a previous study and consensus-based answers to those questions, 22 statements were formulated to test knowledge of participants on OA (and treatment). Each statement could be scored as: “I totally disagree”, “Disagree”, “Agree”, “Totally agree” or “I don’t know”. A total score with a maximum of 22 could be calculated by awarding each correct response with 1 point. Each incorrect or undecided (“I don’t know”) answer was scored as 0 points.

Patient satisfaction was measured directly after finishing the course. Patients were asked how they overall rated the course on a scale from 1 to 10.

### Statistical analyses

Baseline descriptive statistics were calculated as mean and standard deviation (SD), numbers with percentages (%) or median and Interquartile range (IQR). Changes over time in contacts with different healthcare providers were analysed using the exact McNemar’s test and Wilcoxon Signed-Rank test. Difference between baseline and follow-up in secondary outcomes were analysed using the exact McNemar’s test or Paired sample t-tests (two-sided). For all analyses a significance level of *p* ≤ 0.05 was assumed.

## Results

### Patient characteristics

In total 146 patients with knee or hip OA and 54 of their partners participated in the educational program. Overall mean rating of satisfaction with the program was 8.0 (range 1–10). A total of 143 patients agreed to participate in the present study, 107 (75%) participants filled out both questionnaires, 4 were considered drop-outs, as they did not come to the intervention and did not want to continue with the study. Two participants had undergone surgery during the follow-up period and did not feel like to continue. One did have knee OA, but as symptoms of her hand OA were more severe, she did not feel like filling out another questionnaire. All other 29 participants were lost to follow-up without providing a reason. We found no differences on baseline characteristics between drop-out/loss to follow-up and those who completed follow-up questionnaires. Despite the exclusion criteria, 17 participants reported to have had previous joint replacement. Sensitivity analyses showed no differences on HCU regarding surgical visits. Therefore, these participants were not excluded from analysis.

The average age of participants was 69.1 years (SD 10.2), with the majority being female (62.9%). Fifty-six percent of the participants had experienced their OA symptoms for less than 5 years. Patient characteristics are presented in Table 1.

**Table 1:** Baseline demographic and clinical characteristics of participants (*n* = 143)

*Social-demographic characteristics*	
Gender, n (%)	
Female	90 (62.9)
Age *(years),* mean ± SD	69.1 ± 10.2
Ethnicity, n (%)	
Native	131 (91.6)
Living situation, n (%)	
Living together with partner and/or family	102 (71.8)
Level of Education, n (%)	
Low (< 12 years)	90 (64.3)
Work, n (%)	
Paid work	28 (19.7)
District	
1	44 (30.8)
2	44 (30.8)
3	55 (38.5)
*Clinical characteristics*	
Location, n (%)	
Hip	77 (53.9)
Knee	103 (72.0)
Number of painful joints (range 0–10); median (IQR)	3 (2–4)
Duration of symptoms, n (%)	
< 1 years	13 (9.2)
1–5 years	66 (46.8)
5–10 years	32 (22.7)
> 10 years	30 (21.3)
Number of comorbidities (range 0–15); median (IQR)	1 (0–3)

### Healthcare utilization

Table 2 shows the HCU during the 3 months before baseline and during 3-months follow-up. Most common were visits to a physio- or exercise therapist, GP and orthopaedic surgeon regarding knee or hip OA. A significant decrease in proportion of patients who visited the physio- or exercise therapist and GP in the previous 3 months was observed. Although no changes in median number of contacts were seen, the total number of contacts increased. Small but non-significant changes in proportion of patients who visited a medical specialist were found. However, median number of visits to a medical specialist showed a small decrease, which was also seen in the total number of contacts in secondary care.

**Table 2:** Changes in proportion of patients visiting different healthcare providers and total number of contacts with healthcare providers between baseline and 3 months follow-up (*n* = 107)

	*Baseline*	*Follow-up*	
	*Contacted in* *last 3 months* *n (%)*	*Contacted in* *last 3 months* *n (%)*	*p-value* ^*a*^
*Primary care*			
General practitioner	43 (40.2)	27 (25.2)	0.01*
Physio- or exercise therapist	39 (36.5)	26 (24.3)	0.02*
Dietician	3 (2.8)	3 (2.8)	1.00
Occupational therapist	2 (1.9)	–	–
Psychologist	1 (0.9)	–	–
Nurse (in GP practice)	6 (5.6)	5 (4.7)	1.00
District nurse/home care	1 (0.9)	2 (1.9)	–
Total number of contacts Median (IQR)	258 1 (0–2)	327 0 (0–3)	0.48^b^
*Secondary care*			
Rheumatologist	6 (5.6)	3 (2.8)	0.25
Orthopaedic surgeon	20 (18.7)	15 (14.0)	0.30
Physician assistant / nurse practitioner	3 (2.8)	2 (1.9)	1.00
Multidisciplinary team care / pain clinic	1 (0.9)	–	–
Total number of contacts Median (IQR)	46 0 (0–0)	24 0 (0–0)	0.02^b^*

^a^Exact McNemar significance probability

^b^Wilcoxon Signed-Rank test

*Significant for *p*-value ≤0.05

### Secondary outcomes

Changes in secondary outcomes are shown in Table 3. Illness perceptions changed positively (Δ-1.8; 95% CI: 0.4–3.4), and knowledge on OA and treatment options improved (Δ2.4 95% CI: -3.0 - -1.6). No changes in BMI, pain, functioning, self-efficacy and patient activation were found. However, a trend towards an increase in physical activity was seen.

**Table 3:** Differences between baseline and follow-up on secondary outcome measures (*n* = 107 complete cases)

	*Baseline*	*Follow-up*	*p-value*
BMI (kg/m^2^), mean (SD)	27.1 (4.4)	26.7 (4.1)	0.16^b^ *
WOMAC pain (range 0–100), mean (SD)	66.8 (21.4)	69.7 (20.1)	0.13^b^
WOMAC functioning (range 0–100), mean (SD)	68.3 (19.6)	67.8 (21.2)	0.78^b^
Medication use, n (%)			
Paracetamol	65 (61.9)	62 (59.1)	0.65^a^
NSAIDS	33 (32.4)	25 (24.5)	0.08^a^
Other	14 (13.1)	17 (15.9)	0.45^a^
SQUASH Total activity (min/week), mean (SD)	2128.9 (1023.1)	2349.2 (1246.8)	0.07^b^
IPQ Illness perceptions (range 0–100), mean (SD)	41.3 (10.5)	39.5 (10.5)	0.02^b^ *
GSES Self-efficacy (10–40), mean (SD)	32.1 (5.9)	32.2 (5.6)	0.85^b^
PAM-13, patient activation (13–52), mean (SD)	39.3 (0.5)	40.1 (0.5)	0.15^b^
Knowledge on OA (0–22), mean (SD)	10.5 (3.7)	12.9 (3.1)	0.00^b^ *

^a^Exact McNemar significance probability

^b^Paired sample t-test, two-sided

*Significant for *p*-value ≤0.05

## Discussion

Results of the present study show a decreased HCU, the proportion of patients having contact with a physio- or exercise therapist, or general practitioner decreased after following the educational program. We found an increase in knowledge on OA and patients’ perceptions towards their OA changed positively after the course. No significant changes were found in BMI, pain and functioning, physical activity, patient activation and self-efficacy.

Overall, our results are in line with the Cochrane review on self-management programs of Kroon el al. [25]; we also did not find any changes on self-efficacy, pain and functioning. This review however, did not evaluate the effect of self-management programs on illness perceptions, OA knowledge and HCU. We believe that the changes in these parameters are relevant to patients. This is in line with a recent randomized controlled trial, evaluating the effect of a patient decision aid for patients considering joint replacement, (including patient education on treatment options, benefits and risks) that reported positive results on knowledge and illness perceptions [26]. This is important to ensure realistic expectations of treatment outcomes in patients with hip or knee OA, and ultimately, to support self-management in the long-term.

The primary outcome in the evaluation of educational and self-management interventions is under debate [27–29]. In the review by Newman et al. (2004) some included studies used outcomes that are not specifically targeted at the intervention. They concluded that this may decrease the overall effectiveness of educational self-management programs [27]. Similarly, Nolte et al. (2013) argue to critically choose outcome measures which are linked to those targeted for in the intervention, in order to prevent incorrect interpretation of effectiveness [28, 29]. However, in general, multiple outcome dimensions are targeted in self-management interventions. As a result across studies a wide variety of outcome measures is used to evaluate self-management interventions. Usually, pain and/or physical functioning are the primary outcome measures [25]. However, it is questionable whether changes can be expected in these outcomes, when self-management programs are aimed at providing individuals with skills how to cope with symptoms, manage their disease in daily living and navigate the healthcare system [29]. Knowledge on disease management is not the same as changing your behaviour into actually doing it yourself. Therefore, knowledge is often used as a process outcome, and seems more appropriate as secondary outcome. In contrast, HCU is more a measure for behaviour. Based on previous observations that self-management interventions can result in changes in healthcare utilization [29–31] and the assumption that effective self-management ultimately impacts healthcare consumption our choice to explore HCU as primary outcome seems to be appropriate.

In our program we educated patients on what they can do for themselves, when to seek guidance for conservative treatment options and helped them to form realistic thoughts on the expected results of surgical treatment. Following this perspective, changes in HCU patterns could be expected. Our results showed a decrease in patients visiting primary care providers. However, only small non-significant changes in number of patients visiting secondary care specialists were found. Both observations may be explained by the short-term follow-up and small sample of our study. First, as we educated patients on what they can do for themselves (i.e. lifestyle advice on exercise, weight reduction and medication use*)*, some patients may not have felt the need to visit a primary care healthcare provider on short-term, because they directly can put into practice what they have learned during the program [32]. Second, it is possible that patients were already referred to secondary care previous to the intervention, resulting in no short-term changes in secondary care use. Besides, research has shown that education in combination with exercise therapy may postpone surgery in hip OA patients in the long term [33, 34]. This emphasizes the desirability to study long-term results of our educational program in a larger sample.

Remarkably, the total number of contacts in primary care increased whereas the median number of contacts did not change. This finding may reflect the great variability in HCU between participants and specifically the difference in treatment between healthcare professionals. For example, patients will visit their GP once or twice for OA within 3 months, whereas they may visit a physiotherapist once or twice a week. This can sum up to a total of 12–24 visits over 3 months. In the present study several patients started physiotherapy treatment 1–2 weeks prior to the intervention (1–4 visits in the previous 3 months) and continued this treatment after the intervention (> 10 visits in the 3 months post-intervention) (data not shown). This may have contributed to the increased number of total visits in our sample. However, the low number of participants and short-term follow-up of the present study do not allow firm conclusions on this aspect of HCU.

We chose a multidisciplinary approach; in both the developmental process as well as in the execution of the program. This approach is based on previous research which argues to focus on the communication between healthcare providers involved in OA treatment to improve prescription of non-surgical treatment options [9]. In the process of achieving consensus on the content of the program and answering frequently asked questions on OA, we targeted differences in beliefs among healthcare providers regarding the efficacy of non-surgical treatments [9, 35] and clarified roles of different healthcare providers in the management of OA-patients [11]. Consequently, this resulted in clear and consistent information that could be disseminated during the course. This could explain the increased knowledge of patients after participating in the program. So far, little research has been done on the impact of consistency of information on self-management skills across settings and across disciplines for patients with osteoarthritis. In our opinion this is an important area for future research.

We chose to adapt the program to local context and patients preferences as it is known that adapting to local context positively influences knowledge translation [32, 36]. We involved local health care providers in the development and the execution of the program to support the role that health care providers have in patients’ treatment consideration [37] and offering patients options for local support. This may have contributed to the accessibility of the program and may have resulted that our educational program was highly valued by participants (satisfaction score 8 on a scale 1–10).

An important factor in the set-up of our program was the option for participants to bring their partner or a significant other person. Previous studies that focused on explaining reasons for underuse of conservative treatment, underline the importance of the social environment of patients to be involved their care process [9, 37]. Involving a spouse in an intervention may even enhance self-efficacy and improve coping abilities [38], and improve physical activity levels in in OA patients [39]. Our results showed no improvement in self-efficacy after the intervention and only a small, but non-significant increase in physical activity. However, only one-third of the patients who participated in the educational program indeed brought their partner. Future improvements of our intervention should focus on ways to better involve patients’ social environment [9].

This study has several limitations that should be taken into account when interpreting the results. First, the uncontrolled design of the study and the small sample size urges that conclusions drawn about the effect of the intervention should be taken with caution. In our study we examined short-term preliminary effects of a multidisciplinary educational program. However, a controlled trial with long-term follow-up is needed to further explore effects on HCU behaviour in patients with hip or knee OA. Second, we had a 25% loss to follow up, despite reminder letters. The overall high age of our participants might have contributed to the loss. Last, there may be a matter of selection bias. Although we tried to minimize this in our procedure when inviting patients for our study, we have no data available of patients who did not respond to our invitation to participate in our study.

## Conclusions

Our results suggest that a multidisciplinary educational program, may result in changes in HCU and have positive effects on illness perceptions and knowledge in patients with hip or knee OA. These results indicate that patients may better understand and adjust their health seeking behaviour as a result of the program. Especially, the collaboration between health professionals from different disciplines, both in developing and executing the educational program, provides in adequate and consistent information on OA, treatment and self-management options. A randomized controlled trial with long-term follow-up with larger number of patients is needed to confirm these results.

## Additional file

Additional file 1: A detailed description of the process of inventory and prioritising of FAQs. (DOCX 18 kb)

## Abbreviations

BMI: body mass index
FAQ: frequently asked question
GP: general practitioner
GSES: General Self-efficacy Scale
HCU: healthcare utilization
IPQ: Illness perceptions questionnaire
NSAID: Non-steroidal anti-inflammatory drug
OA: osteoarthritis
SQUASH: Short Questionnaire to Asses physical activity
WOMAC: Western Ontario McMaster University Index of osteoarthritis
