# Supplementary material for: Preliminary effects of a regional approached multidisciplinary educational program on healthcare utilization in patients with hip or knee osteoarthritis: an observational study
Source: BMC Fam Pract. 2018 Jun 6;19:82. doi: 10.1186/s12875-018-0769-7 (PMC5991450; doi:10.1186/s12875-018-0769-7)
Supplement: Supplementary file 1 — A detailed description of the process of inventory and prioritising of FAQs. (DOCX 18 kb) [file 12875_2018_769_MOESM1_ESM.docx]

**Additional file 1 – Process of inventory and prioritising of frequently asked questions (FAQs)**

A five-step systematic approach was taken to determine important information that needed to be included in the educational program.

**Step 1 - Inventory**

An inventory of FAQs about OA was made among local health care providers. FAQs were collected among: 1) specialists from the Departments of Orthopaedic Surgery and Departments of Rheumatology in three local hospitals, 2) GP’s involved in the project, as well as GP’s within their network, 3) physiotherapists from local OA-networks, 4) the Dutch Arthritis Foundation. All health care providers were asked to record 5-10 FAQs they often get from their OA-patients, of which the answers are not covered on the website [www.thuisarts.nl](http://www.thuisarts.nl) (Nederlands Huisartsen Genootschap, n.d.). This website already covers general information about OA, based on national and international guidelines. A total of 192 questions were collected among 28 health care providers. After deleting overlapping ones and questions covered by the before mentioned website, 99 FAQs remained for the prioritising step.

**Step 2 – Categorising**

The 99 FAQs were categorised into 9 categories by to researchers independently (7-14 FAQs per category). Categories were: “OA, cause, consequences and disease course”, “diagnostics”, “medication”, “health care providers”, “lifestyle”, “surgery”, “work”, “self-management”, “other”.

**Step 3 – Prioritising**

Health care providers who provided FAQs were asked to indicate a top 5 most important FAQs per category and divide 100 point among their top 5. Moreover, they were asked to prioritise the categories, with regard to importance for an educational program for patients with OA. Additionally, a call for patients with OA willing to prioritise those FAQs by patients with OA, was placed on several websites, a local OA-network website as well as on the website of a non-profit foundation, covering all patient organisations in the Netherlands (e.g. “Stichting Reumazorg Nederland”), and the websites of two local physiotherapy practice’s. The same prioritising method used for health care providers, was used among patients with OA. From the 5 highest ranked categories the 3 highest ranked scored FAQs were included. From the other 4 categories the 2 highest ranked FAQs were included. Based on overall highest scores, 7 more FAQs were added to complete a Top 30 of most important FAQs. This Top 30 was used for answer formulation.

**Step 4 – Answering FAQs**

An expert group was formed by 2 orthopaedic surgeons, 1 rheumatologist, 1 specialized nurse, 3 physiotherapists, 1 general practitioner and 1 researchers. Each member of the expert group answered 10 FAQs. They were asked to formulate answers as they would when answering a patients. Because every expert answered 10 FAQs, every FAQ was answered three times.

Step 5 – Formulating definite answers

In 3 consensus meetings with the expert group, answers were discussed and combined into one final answer per FAQ. Final answers were edited by a communication specialist.
